# Characterizing the Feeding Injury Caused by *Phylloscelis rubra* (Hemiptera: Dictyopharidae) to Cranberries

**DOI:** 10.1093/jisesa/ieaa143

**Published:** 2020-12-25

**Authors:** Cesar Rodriguez-Saona, Vera Kyryczenko-Roth, Daniel Schiffhauer, Nicolas Firbas

**Affiliations:** 1 P.E. Marucci Center, Rutgers University, Chatsworth, NJ; 2 Ocean Spray Cranberries, Chatsworth, NJ; 3 Departamento de Matemática Aplicada, Universidad de Valencia/Universidad Politécnica de Valencia, Valencia, Spain

**Keywords:** cranberry toad-bug, pest management, vine injury, fruit injury, nutrient content

## Abstract

Due to changes in pest management practices, farmers’ reports of severe feeding injury to cranberries, *Vaccinium macrocarpon* Aiton Ericales: Ericaceae, caused by the cranberry toad-bug, *Phylloscelis rubra* Ball, have increased in recent years in New Jersey (United States). Currently, however, limited information is available on the effects of *P. rubra* feeding or density of individuals needed to cause injury to cranberry vines and fruit. In 2015‒2017, we conducted studies to characterize injury to cranberry at a range of *P. rubra* densities by using cages in a screen-house and field, to establish a correlation between *P. rubra* density and crop injury in an open field experiment, and to measure the effects of *P. rubra* injury on the nutritional content (i.e., amounts of macro- and microelements) of cranberry vines. *Phylloscelis rubra* feeding on cranberry vines produced typical injury symptoms at relatively low densities (i.e., 2 individuals per vine in field cages or <10 individuals per sweep net sample in open fields), which included discolored (yellowish or reddish) or dead (brown) vines. This vine injury could lead to reductions in fruit mass and total fruit number. However, *P. rubra* injury to cranberry vines did not alter their nutritional composition. In general, this study highlights the ability of *P. rubra* to cause substantial injury to cranberry vines even when population densities were relatively low, which could result in declines in fruit production (quality and quantity). Therefore, infestations by *P. rubra* in cranberries must be considered when making pest management decisions in regions where this insect is present.

Management practices for insect pests of agricultural crops are constantly adapting to regulatory changes on insecticide use (Zalom 2010, Rodriguez-Saona et al. 2014). For example, in the United States, several broad-spectrum insecticides, i.e., organosphates and carbamates, have been banned or restricted for use following the implementation of the Environmenal Protection Agency (EPA) Food Quality Protection Act of 1996 (EPA FQPA 1996). These changes in insecticide use have affected all crops in the United States (e.g., Jones et al. 2010) but particularly those that are not widely grown (so-called specialty or minor crops that are grown on less than a total of 121,410 hectares for the crop; Holm and Kunkel 2004) and have a short list of registered insecticides, such as is the case of the American cranberry (*Vaccinium macrocarpon* Aiton).

The American cranberry is a perennial low-growing woody vine that is native to acidic, sandy soils of northeastern North America and grows in beds maintained on farms (also known as bogs or marshes). The United States is the world’s largest producer of cranberries, with Wisconsin, Massachusetts, New Jersey, and Oregon being the highest cranberry-producing states (NASS-USDA 2019). Historically, three lepidopteran insects have been considered major pests of cranberries in these regions: Sparganothis fruitworm [*Sparganothis sulfureana* (Clemens) (Lepidoptera: Tortricidae)], blackheaded fireworm [*Rhopobota naevana* (Hübner) (Lepidoptera: Tortricidae)], and cranberry fruitworm [*Acrobasis vaccinii* Riley (Lepidoptera: Pyralidae)] (Averill and Sylvia 1998, Gallardo et al. 2018). Although these lepidopteran species are still considered significant pests of cranberries across the United States, their ranking in importance has slightly changed in recent years due to new insect pest outbreaks as a result of changes in management practices.

In New Jersey, cranberries constitute one of the major fruit crops in the state with a total annual production in 2018 of 23.2 million kg valued at US$15.8 million (NASS-USDA 2019). Following restrictions on broad-spectrum insecticides by the EPA FQPA, use of narrow-spectrum, reduced-risk insecticides increased in cranberries in the 2000s, in particular methoxyfenozide (Intrepid; Corteva Agriscience, Indianapolis, IN), for the control of lepidopteran pests, such as *S. sulfureana* and *R. naevana*. However, methoxyfenozide has no control over piercing-sucking insects. As a result, cranberry farmers in New Jersey who adopted these narrow-spectrum insecticides experienced effective control of lepidopteran (chewing) pests, but had outbreaks of other insects with different feeding habits, such as the native cranberry toad-bug (*Phylloscelis rubra* Ball) (McPherson and Wilson 1995, Bartlett et al. 2011), that were rarely seen in high abundance or causing economic injury.

In 2013, after almost 100 yr of *P. rubra* not being reported as an important pest of cranberries (Sirrine and Fulton 1914), we observed severe injury to vines caused by *P. rubra* in a commercial cranberry farm in Chatsworth, New Jersey. Although we had occasionally seen *P. rubra* on cranberry beds in the past, we had never seen them causing serious injury to the vines and fruit. Because of its rare occurrence as a pest, little is known about the biology and potential economic effects of *P. rubra* on cranberries. *Phylloscelis rubra* is a specialist on cranberries (Sirrine and Fulton 1914), whose distribution in the United States ranges from New York south to Florida and west to Mississippi (Ball 1930). According to Sirrine and Fulton (1914), in New York, this insect has one generation a year and overwinters as eggs. The nymphs appear by the end of June through August and the adults from early August through October; eggs are laid from September through October. Feeding injury to cranberry vines, as described by Sirrine and Fulton (1914), causes closing in (toward the branch) of the leaves on the new growth (first stage) and then changes in color (reddish to brown) of the new growth (second stage). In New Jersey, feeding injury by nymphs and adults can be seen from July until harvest (i.e., October; C.R.-S. personal observation). Severe injury can cause vines to die and the berries to shrivel up, resulting in dwarfed berries and reduced yield (Sirrine and Fulton 1914).

Currently, we lack knowledge regarding the relationship between *P. rubra* density and injury to cranberry vines and fruit. We hypothesized a positive relationship between *P. rubra* density and injury to vines and a negative relationship between their density and crop yield. To test this hypothesis, we conducted a 3-yr study (2015‒2017) to 1) determine the effects of a range of *P. rubra* densities on injury to cranberries in cages under a screen-house (i.e., potted plants) and open field conditions, 2) establish a relationship between *P. rubra* densities and injury to vines and fruit in commercial cranberry beds, and 3) measure the effects of *P. rubra* injury on the nutritional content of cranberry vines. Information on the effects of *P. rubra* feeding injury to cranberries is important to help establish density thresholds and reduce economic losses by this emerging pest.

## Materials and Methods

### Insects

*Phylloscelis rubra* adults used in experiments were collected during mid-to-end of July of 2015, 2016, and 2017 from infested cranberry beds at a commercial cranberry farm in southern New Jersey (Pine Island Cranberry Company Inc., Chatsworth, NJ) by using a sweep net. We used adults because we expect this stage to be the most damaging to cranberries. Adults were collected on the same day of the experiments and placed in a small white cage (30 cm × 30 cm × 30 cm, ‘BugDorm’, catalog no. 1452; BioQuip Products, Rancho Dominguez, CA) in the laboratory (~22°C) until needed.

### Screen-House Cage Study

**Fig. 1.**
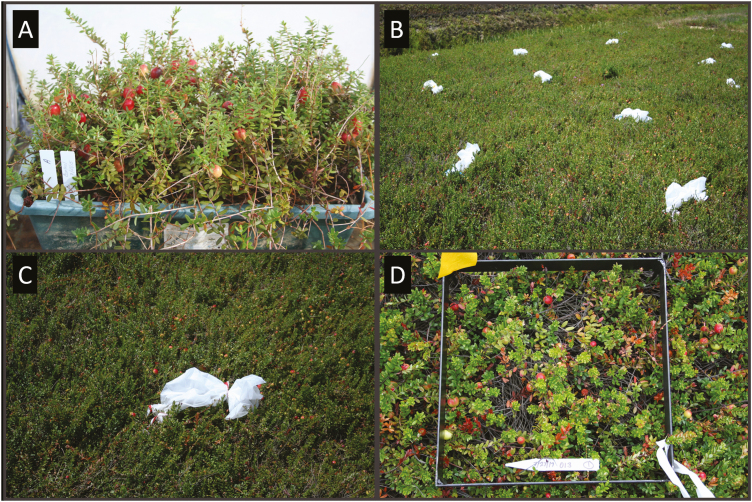
Example of a tray (43.2 cm × 19.1 cm × 16.5 cm) covered with 2-yr-old cranberry (*Vaccinium macrocarpon*) vines that was placed inside large white cages (61 cm × 61 cm × 91.4 cm) in an outdoors screened cold frame (screen-house) (A). Fiber sleeve cages (20.3 cm × 40.6 cm × 50.8 cm) containing five young vines and fruit in a cranberry bed (B). Close up of a fiber sleeve cage (C). Metal (0.3 m^2^) frame next to a yellow flag (upper left corner) used to assess *Phylloscelis rubra* injury to vines and fruit in cranberry beds (D).

A study was conducted in 2015 to determine the effects of *P. rubra* density on injury to potted cranberry plants containing vines with leaves and fruit. The study was performed in large white cages (61 cm × 61 cm × 91.4 cm, ‘Popup Rearing & Observation Cages’, catalog no. 1466CV; BioQuip Products) placed outdoors in a screened cold frame (screen-house). A 43.2 cm × 19.1 cm × 16.5 cm tray covered with 2-yr-old cranberry (*V. macrocarpon* ‘Stevens’) vines (Fig. 1A) was placed in the center of each cage. Because *P. rubra* injures vines when fruit is developing, before the experiment, the boxes were placed outdoors for about a month for flowers to be pollinated and the fruit to start developing to mimic field conditions. *Phylloscelis rubra* adults (mixed sexes at roughly 1:3 female:male ratio, as seen in the field; C.R-S., personal observation) were placed inside the cages on 23 July at four densities: 0 (control), 10 (‘low’ density or ~0.02 adults/vine), 25 (‘medium’ density or ~0.05 adults/vine), and 50 (‘high’ density or ~0.1 adults/vine) per cage. The experiment was a complete randomized block design with each insect density replicated five times (blocks), with each block containing one of the treatments (*n* = 20 cages), to avoid potential effects due to environmental variation, such as temperature and light intensity, within the screen-house. Plants inside boxes were watered daily for about 0.5 h with the aid of an automatic sprinkler system. After 7‒8 wk (on 9‒18 September), each box was taken to the laboratory for evaluation; this time frame covers most of the adult feeding period on cranberries and allowed fruit to complete maturation. In the laboratory, all vines in each box (replicate) were cut and evaluated for total number of healthy vines and vines injured due to stress or other unknown causes. Vines were classified as ‘healthy’ if they had mostly green leaves with no signs of injury, ‘stressed’ if at least 25% of the leaves were discolored (yellowish or reddish) or dead (brown; a sign of *P. rubra* injury according to Sirrine and Fulton 1914, V.-K-R., personal observation), and ‘other’ if vines had leaves injured by unknown causes. In addition, we recorded the number and weight of ‘healthy’, ‘rotten’, and ‘other’ fruit. Fruit was classified as 'rotten' if it had obvious signs of decay (possibly as a result of *P. rubra* injury to vines based on Sirrine and Fulton 1914) or 'other' if fruit had injury marks made by unknown causes; otherwise, all marketable fruit was placed in the 'healthy' category.

### Field Cage Study

A study was conducted in 2016 to determine the effects of *P. rubra* density on injury to cranberry vines and fruit under field conditions. The experiment was performed in an experimental cranberry (*V. macrocarpon* ‘Stevens’) bed at the Rutgers P.E. Marucci Center (Chatsworth, New Jersey). Cages consisted of five new, young vines enclosed in 20.3 cm × 40.6 cm × 50.8 cm fiber sleeves (Temkin International Inc., Payson, UT; Fig. 1B and C) on 18 July; thus, besides leaves, vines contained young (green) fruit. There were four density treatments, namely, 0 (control), 2 (low density), 5 (medium density), and 10 (high density) *P. rubra* adults (mixed sexes, 1:3 female:male ratio), such that low = fewer than 1 adult/vine, medium = 1 adult/vine, and high = 2 adults/vine. The experiment was a randomized complete block design, with 20 cages (five cages per treatment) within a block and each cage separated at least 2 m from each other. Blocks were replicated four times (*n* = 20 cages per treatment) and were at least 20 m apart. After setup, adult *P. rubra*, at different densities depending on the treatment, were placed inside the cages and kept there until 16 August (4 wk after infestation), when all cages with their contents were removed from the field and brought to the laboratory for examination. In the laboratory, for each cage (replicate), we counted the total number of healthy, stressed, and other vines, as described above. We also recorded the number and weight of healthy, rotten, and other fruit, as previously described.

### Field Injury Assessment

A study was conducted in 2017 in commercial cranberry beds to correlate *P. rubra* density and vine and fruit injury under field conditions. The number of *P. rubra* was monitored in seven cranberry (*V. macrocarpon* ‘Stevens’) beds (~1‒2 ha each): five beds were located at a commercial cranberry farm (Pine Island Cranberry Company Inc.) and two beds at the Rutgers P.E. Marucci Center. These beds were selected because of their previous history of high *P. rubra* infestation. All beds were divided into three equal-size sections: two sections corresponded to both ends of the bed and one in the middle of the bed. Each section was marked by a colored flag in the middle, and samples were taken with a sweep net (30.5 cm diameter; BioQuip Products) by walking a straight line crossing the section. Each sweep net sample started at least 10 m from the bed’s edge and consisted of 25 sweeps according to Averill and Sylvia (1998). Thus, three sweep net samples (one sample per section) were taken in each of the seven beds weekly from 26 June until 18 September (total of 13 sampling dates). The same sections of the bed were sampled on each sampling date, and samples were placed in individual bags labeled by bed number, section number, and date and were brought to the laboratory for evaluation. The number of *P. rubra* nymphs and adults per sweep net sample was recorded using a stereomicroscope.

To assess injury to vines and fruit, once a month (on 24 July, 14 August, and 4 September), a 0.3-m^2^ metal frame (Fig. 1D) was placed next to the flag where the sweep net samples were taken. The frame was placed in a way that the flag was in its upper left corner (see yellow flag in Fig. 1D) to ensure that the same area was evaluated on each sampling date. Within each frame, the number of healthy and stressed vines was recorded and used to calculate the proportion of stressed vines as the number of stressed vines divided by the number of healthy plus stressed vines. In the final evaluation date, all fruit within the frame was removed and brought back to the laboratory, and the proportion of insect-injured fruit and total number of fruit were recorded as a proxy of fruit quality and quantity (i.e., yield), respectively. The proportion of insect-injured fruit was calculated as the number of insect-injured fruit divided by the total number of fruit.

### Nutrient Analysis

In 2017, we conducted a study to investigate whether *P. rubra* infestation affects nutrient (macro- and microelements) composition in cranberry leaves. The study was conducted in a cranberry (*V. macrocarpon* ‘Stevens’) bed at the Rutgers P.E. Marucci Center. Five new, young vines were enclosed in 20.3 cm × 40.6 cm × 50.8 cm fiber sleeve cages (Temkin International Inc.) on 20 July, for a total of 10 cages. Five of the cages received 10 *P. rubra* adults (i.e., 2 adults/vine; mixed sexes, 1:3 female:male ratio)—this density was used because previous studies showed a negative effect on cranberries (see Results)—and the other five cages received no insects (controls). On 10 August, all vines from each cage were collected for analysis of mineral elements in leaves, placed in separate paper bags, dried at room temperature for about 21 d, and then sent to the Agricultural Analytical Services Laboratory at Pennsylvania State University (University Park, PA; http://agsci.psu.edu/aasl/plant-analysis/plant-methods) for analyses. Nitrogen content was measured by combustion using the Elementar Vario Max C/N Analyzer (Horneck and Miller 1998). Five macroelements (phosphorus [P], potassium [K], calcium [Ca], magnesium [Mg], and sulfur [S]) and seven microelements (manganese [Mn], zinc [Zn], iron [Fe], copper [Cu], boron [B], sodium [Na], and aluminum [Al]) were measured via acid digestion by inductively coupled plasma emission spectroscopy (Huang and Schulte 1985).

### Data Analyses

For both cage studies (screen-house and field), we calculated the percentage of healthy, stressed, and other vines and the percentage of healthy, rotten, and other fruit per cage. We tested the effects of *P. rubra* density (fixed effect) and block on the percentage of vines and fruit for each category by using analysis of variance (ANOVA; Minitab ver. 17, Minitab, Inc. 2010). A significant ANOVA test was followed by Tukey’s pairwise comparisons. Data on the effects of *P. rubra* density on healthy and rotten fruit mass were also analyzed using ANOVA followed by Tukey’s tests, if significant; the mass of other fruit was not analyzed statistically because the number of fruits under this category was too low to conduct meaningful analysis. Data were checked for normality (Anderson-Darling test) and equal variances (Levene’s test) before analysis. If needed, data on fruit mass were ln(*x* + 0.5)-transformed to meet ANOVA assumptions. Percent data were arcsine square-root-transformed before analyses.

In the open field study, the effects of *P. rubra* density (i.e., numbers per sweep net sample) on the proportion of stressed vines, the proportion of insect-injured fruit, and total number of fruit were analyzed using linear mixed model fit by restricted maximum likelihood (R software version 3.4.3; R Core Team 2017). We used the ‘lmerTest’ package, which uses the Satterthwaite’s method, on R software to approximate the *t* values and df (Kuznetsova et al. 2017). In addition, we used regression analysis to correlate *P. rubra* density with the proportion of stressed vines and the total number of fruit.

Principal component analysis (PCA; Minitab Inc. 2010) was performed to visualize differences in the composition of macro- and microelements between healthy and *P. rubra*-injured cranberry leaves. In addition, multivariate analysis of variance (MANOVA) was used to test differences in overall amounts of macro- and microelements between healthy and *P. rubra*-injured cranberry leaves. Following MANOVA, we conducted ANOVA to test for differences in individual macro- and microelements between healthy and *P. rubra*-injured cranberry leaves. If needed, data were ln-transformed to meet ANOVA assumptions before analysis; percent data were arcsine square-root-transformed.

## Results

### Screen-House Cage Study

**Fig. 2.**
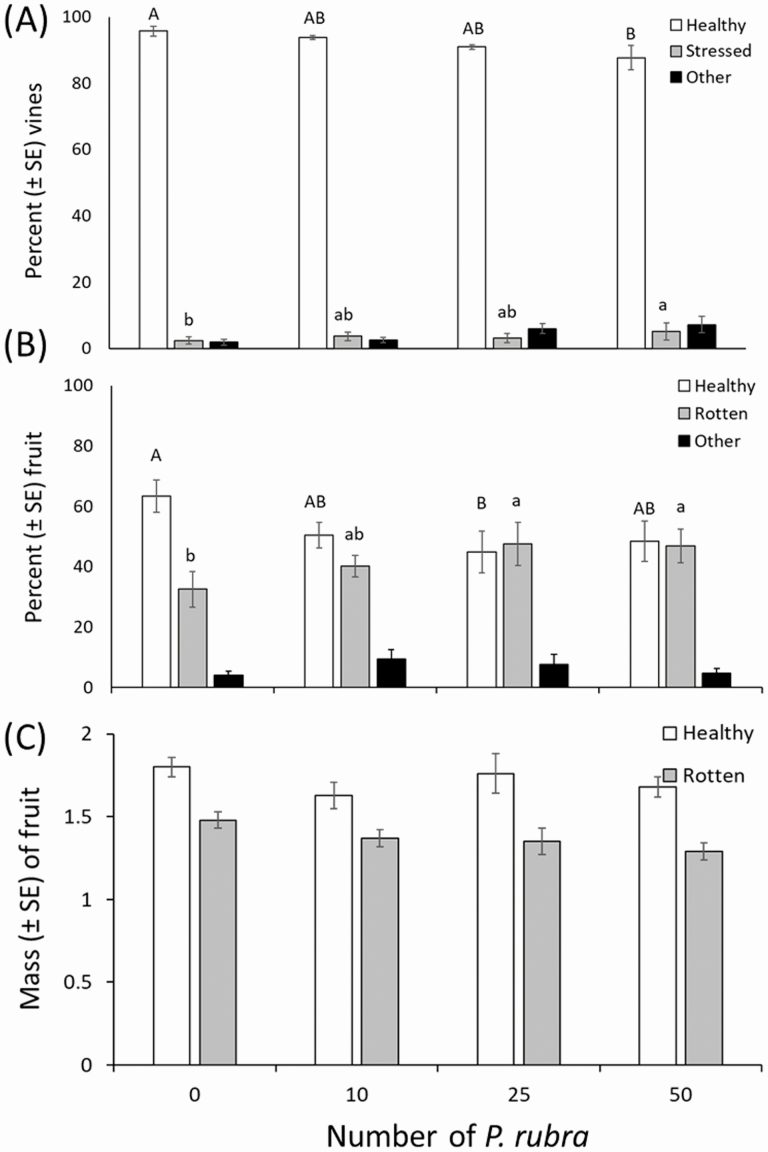
Effects of *Phylloscelis rubra* injury to cranberry vines and fruit in large cages in a screen-house. Mean (± SE) percentage of healthy vines (i.e., vines with mostly green leaves with no signs of injury), stressed vines (i.e., vines with at least 25% discolored or dead leaves; a sign of *P. rubra* injury), and other injured vines (i.e., vines with leaves injured by unknown causes) as a function to *P. rubra* density (A). Mean (± SE) percentage of healthy fruit (i.e., all marketable fruit), rotten fruit (i.e., fruit with obvious signs of decay possibly due to *P. rubra*), and other injured fruit (i.e., fruit with injury marks made by unknown causes) (B). Mean (± SE) mass of healthy and rotten fruit (C). Means with different letters are significantly different, and the means separation test was applied separately to each vine and fruit injury class (Tukey’s post hoc test, *P* ≤ 0.05); the uppercase letters correspond to differences in healthy vines or fruit, whereas lowercase letters correspond to differences in stressed vines or rotten fruit among *P. rubra* density treatments.

The total number of cranberry vines in each box did not differ among treatments (mean ± SE = 497.6 ± 20.34; *F* = 1.79; df = 3, 12; *P* = 0.203). However, there were 8% less healthy vines in the high (50 adults per cage) *P. rubra* density treatment than in the control (0 adults per cage) treatment (*F* = 3.80; df = 3, 12; *P* = 0.04; Fig. 2A). There was also a 2.7% higher percentage of stressed vines in the high-density treatment than the control (*F* = 4.63; df = 3, 12; *P* = 0.023; Fig. 2A). Treatments had no effect on the percentage of vines injured by causes other than *P. rubra* feeding (*F* = 0.69; df = 3, 12; *P* = 0.573; Fig. 2A).

The total number of cranberry fruit in each box did not differ among treatments (mean ± SE = 79.2 ± 5.24; *F* = 1.58; df = 3, 12; *P* = 0.245). However, the percentage of healthy fruit was 18.5% lower in the medium (25 adults per cage) *P. rubra* density treatment than in the control (0 adults per cage) treatment (*F* = 3.90; df = 3, 12; *P* = 0.037; Fig. 2B). The percentage of rotten fruit was ~15% higher in the medium and high *P. rubra* density treatments than in the control (*F* = 4.76; df = 3, 12; *P* = 0.021; Fig. 2B). There were no differences in the numbers of fruit with unknown (other) injury (*F* = 1.00; df = 3, 12; *P* = 0.427; Fig. 2B). There were also no differences among treatments in the mass of healthy (*F* = 1.42; df = 3, 12; *P* = 0.286) or rotten (*F* = 2.96; df = 3,12; *P* = 0.075) fruit (Fig. 2C).

### Field Cage Study

**Fig. 3.**
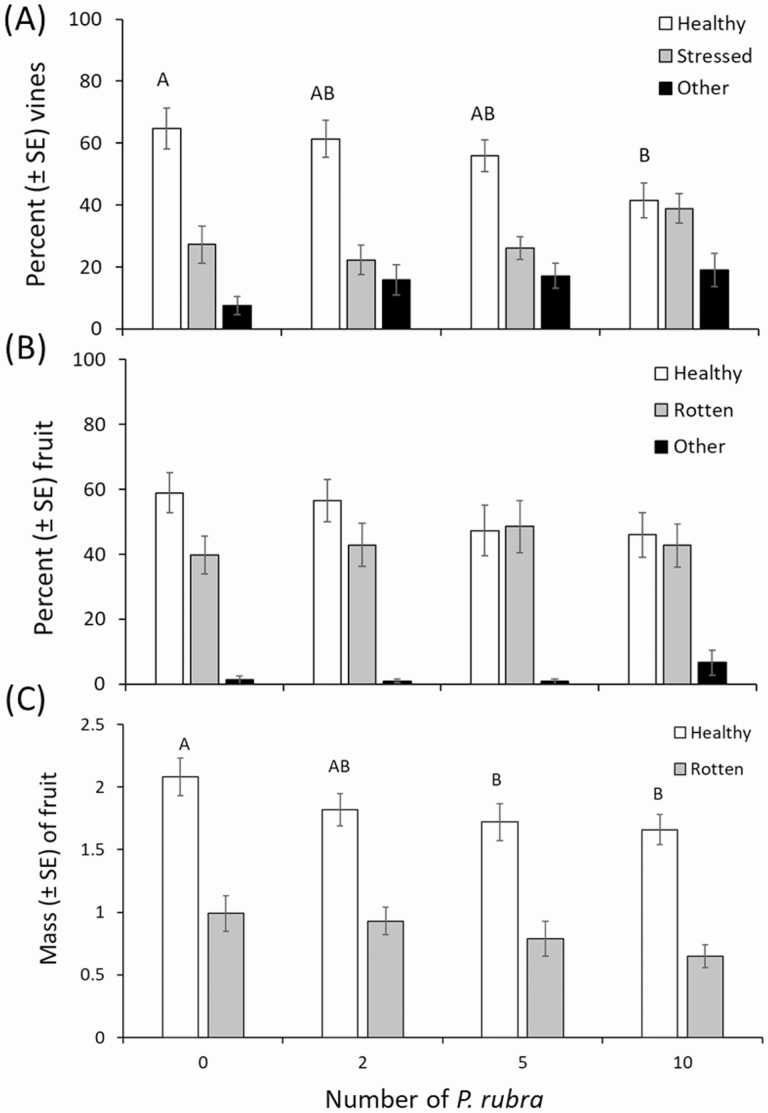
Effects of *Phylloscelis rubra* injury to cranberry vines and fruit in fiber sleeve cages in a cranberry bed. Mean (± SE) percentage of healthy, stressed, and other injured vines (A). Mean (± SE) percentage of healthy, rotten, and other injured fruit (B). Mean (± SE) mass of healthy and rotten fruit (C). See Fig. 2 legend for a description of each vine and fruit injury classification. Means with different letters are significantly different, and the means separation test was applied separately to each vine and fruit injury class (Tukey’s post hoc test, *P* ≤ 0.05).

**Fig. 4.**
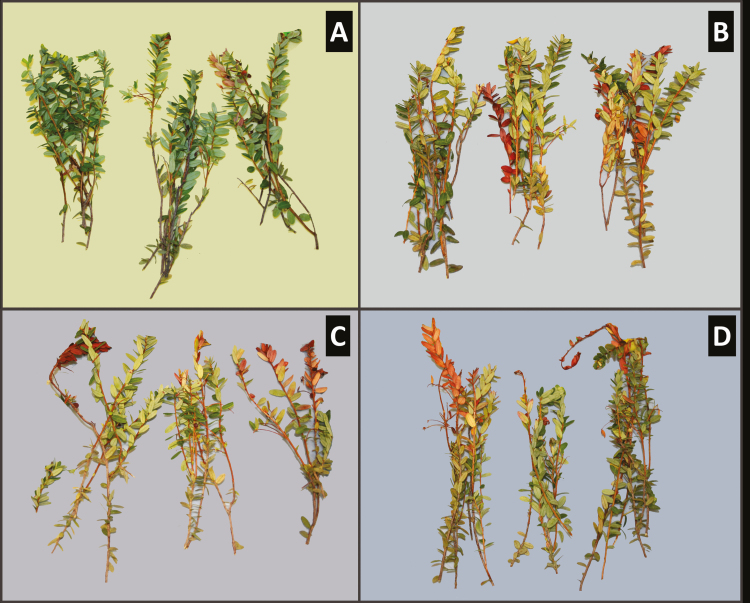
Typical feeding injury by *Phylloscelis rubra* showing discoloration and death of cranberry vines at increasing adult densities: 0 adult/vine (A), a low density of 0.4 adult/vine (B), a medium density of 1 adult/vine (C), and a high density of 2 adults/vine (D).

There were ~23% less healthy vines in the high *P. rubra* density treatment (i.e., 2 adults per vine) than in the control (0 adults per vine) treatment (*F* = 3.57; df = 3, 73; *P* = 0.018; Figs. 3A and 4). However, there were no differences among treatments in the percentage of stressed vines (*F* = 2.36; df = 3, 73; *P* = 0.078; Fig. 3A) and vines injured by causes other than *P. rubra* feeding (*F* = 1.73; df = 3, 73; *P* = 0.169; Fig. 3A).

The total number of cranberry fruit in each fiber sleeve cage did not differ among treatments (mean ± SE = 7.86 ± 0.39; *F* = 2.20; df = 3, 73; *P* = 0.095). There were no differences among treatments in the percentage of healthy (*F* = 0.87; df = 3, 73; *P* = 0.459) or rotten (*F* = 0.36; df = 3, 73; *P* = 0.781) fruit or fruit with unknown (other) injury (*F* = 1.40; df = 3, 72; *P* = 0.249; Fig. 3B). However, the mass of healthy fruit was ~20% lower in the medium (i.e., one adult per vine) and high (two adults per vine) *P. rubra* density treatments than in the control (0 adults per vine) treatment (*F* = 2.94; df = 3, 73; *P* = 0.039; Fig. 3C). There were no differences among treatments in the mass of rotten fruit (*F* = 1.01; df = 3, 73; *P* = 0.392; Fig. 3C).

### Field Injury Assessment

**Fig. 5.**
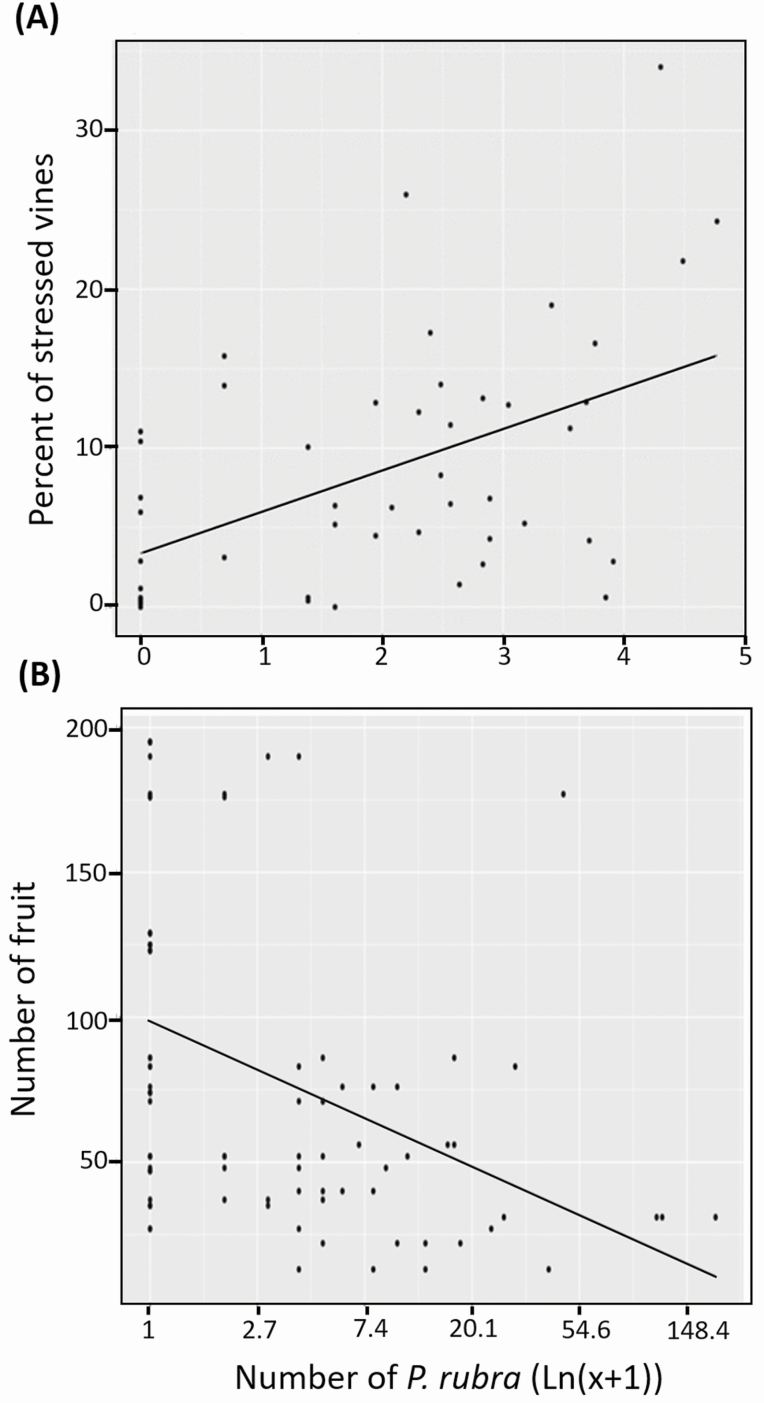
Relationship between the numbers of *Phylloscelis rubra* individuals per sweep net sample and the percentage of stressed vines (A) or the number of fruit (B). Because these periods were critical for *P. rubra* injury to cranberries, data from 24 July and 14 August were used for (A) and weekly data from 3 to 31 July were used for (B). The data were plotted following ln(*x* + 1) transformations, and linear regression lines fitted to the data are shown.

We found a significant effect of *P. rubra* density (numbers per sweep net samples) on the proportion of stressed (injured) vines on 24 July (*t* = 2.39; df = 21; *P* = 0.027) and 14 August (*t* = 3.98; df = 21; *P* < 0.001). There was a significant positive correlation between *P. rubra* density and the percentage of stressed vines (*F* = 14.28; df = 1,46; *P* < 0.001; *Y* = 3.38 + 2.61(ln(*X* + 1)), where *Y* = percent of injured vines and *X* = *P. rubra* density; *R*^2^ = 0.24; Fig. 5A). According to this regression equation, averages as low as five *P. rubra* per sweep net sample could equate to approx. 1.4-fold increase in vine injury. *Phylloscelis rubra* density did not affect the proportion of stressed vines on 4 September (*t* = 0.14; df = 21; *P* = 0.891).

There was a significant effect of *P. rubra* density on the proportion of insect-injured fruit in only one (21 August) out of the 13 sampling dates. There was also a significant effect of *P. rubra* density on the total number of fruit in 4 of the 13 sampling dates (Table 1). The density of *P. rubra* before 31 July was significantly negatively correlated with the total number of fruit (*F* = 17.23; df = 1,92; *P* < 0.001; *Y* = 98.67 − 16.7(ln(*X* + 1)), where *Y* = total number of fruit and *X* = *P*. rubra density; *R*^2^ = 0.16; Fig. 5B). According to this regression equation, even averages of five *P. rubra* per sweep net sample could equate to approximately a 30% reduction in yield.

**Table 1.** Results from linear mixed models for the effects of *Phylloscelis rubra* infestation on the percent of injured fruit and the number of fruit in commercial cranberry beds

Date	Percent injured fruit			No. of fruit		
	*t*-value	df	*P* value	*t*-value	df	*P* value
26 June	0.936	8.5	0.375	1.379	16.1	0.187
3 July	0.643	17.3	0.529	2.132	19.1	**0.046**
10 July	1.986	16.9	0.063	3.352	21.0	**0.003**
17 July	0.819	19.7	0.422	2.980	21.0	**0.007**
24 July	−0.099	21.9	0.922	1.290	21.0	0.211
31 July	0.740	21.8	0.467	2.297	21.3	**0.032**
7 Aug.	0.655	18.5	0.521	1.242	21.1	0.228
14 Aug.	1.135	14.0	0.275	0.924	21.0	0.366
21 Aug.	2.623	20.1	**0.016**	1.161	21.3	0.259
28 Aug.	0.446	15.9	0.662	0.473	21.3	0.641
4 Sept.	−1.211	13.4	0.247	0.116	21.4	0.909
11 Sept.	−0.299	17.8	0.768	0.815	21.1	0.424
18 Sept.	−0.846	16.7	0.409	1.711	21.1	0.102

### Nutrient Analysis

**Fig. 6.**
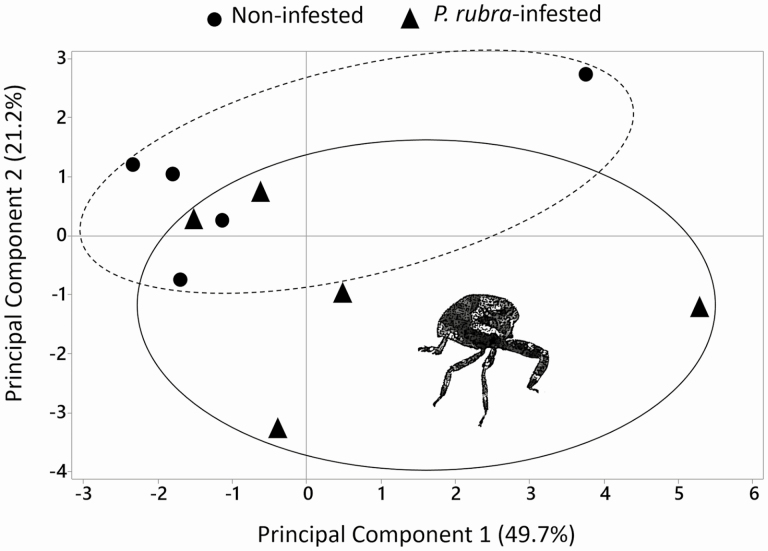
Principal component analysis (PCA) for the effects of *Phylloscelis rubra* infestation on the nutrient composition of cranberry vines. First and second components are plotted against each other, and the percentage variation is explained between parentheses. Each black triangle and black circle represents an individual sample of *P rubra*-infested and noninfested vine, respectively, and all samples for each of the two treatments are enclosed within the ellipsoids. *n* = 5.

The PCA revealed little to no separation in nutrient levels between *P. rubra*-infested and noninfested cranberry plants, with the first two components explaining 70.9% of the variation (Fig. 6). As a group, there was no differences in macronutrient (Wilks’ lambda = 0.365; *F* = 0.87; df = 6, 3; *P* = 0.597) or micronutrient (Wilks’ lambda = 0.212; *F* = 1.07; df = 7, 2; *P* = 0.565) nutrient composition between *P. rubra*-infested and noninfested cranberry leaves. Individually, there were no significant differences in any macro- or micronutrients between *P. rubra*-infested and noninfested cranberry plants (Table 2). Altogether, these data do not provide evidence that *P. rubra* feeding injury alters the nutritional quality or quantity of cranberry leaves.

**Table 2.** Effects of *Phylloscelis rubra* infestation on the levels of macro- and micronutrients in cranberry vines^*a*^

Nutrients	Mean ± SE		*F*	df	*P* value
	*P. rubra*-infested	Noninfested			
Nitrogen^*b*^	1.19 ± 0.05	1.06 ± 0.03	4.41	1,8	0.069
Phosphorus^*b*^	0.16 ± 0.007	0.15 ± 0.009	0.19	1,8	0.671
Potassium^*b*^	1.10 ± 0.15	0.89 ± 0.10	1.06	1,8	0.344
Calcium^*b*^	0.17 ± 0.05	0.24 ± 0.08	0.64	1,8	0.447
Magnesium^*b*^	0.07 ± 0.02	0.07 ± 0.01	0.06	1,8	0.816
Sulfur^*b*^	0.09 ± 0.004	0.09 ± 0.005	0.08	1,8	0.782
Manganese^*c*^	138.22 ± 44.31	199.21 ± 77.53	0.39	1,8	0.548
Iron^*c*^	185.78 ± 18.65	249.48 ± 27.79	2.90	1,8	0.127
Copper^*c*^	4.76 ± 0.28	4.79 ± 0.57	0.01	1,8	0.906
Boron^*c*^	23.62 ± 2.41	28.28 ± 3.12	1.12	1,8	0.322
Aluminum^*c*^	90.34 ± 6.43	123.04 ± 12.12	4.54	1,8	0.066
Zinc^*c*^	24.99 ± 1.13	25.20 ± 0.59	0.02	1,8	0.882
Sodium^*c*^	63.92 ± 7.19	55.52 ± 8.81	0.44	1,8	0.528

^*a*^Samples (*n* = 5 per treatment) were taken on 10 Aug. 2017.

^*b*^Macronutrients. Data are in percentages.

^*c*^Micronutrients. Data are in mg/kg.

## Discussion

Little is known about the type of feeding injury caused by *P. rubra* to cranberries and its potential implications for fruit production. This study is the first systematic evaluation of injury by *P. rubra* feeding on cranberries and demonstrates that feeding injury by this insect can potentially cause discoloration and death of cranberry vines. Although this injury to vines did not alter their nutritional content (i.e., number or amount of nutrients), under certain conditions, it can result in the reduction of fruit quality (mass) and quantity (total yield). Besides *P. rubra*, we are unaware of other species in the family Dictyopharidae (Hemiptera) that cause economic injury to crops. However, *Taosa longula* Remes Lenicov (Hemiptera: Dictyopharidae) has been considered a candidate for the biological control of an aquatic weed, the water hyacinth *Eichhornia crassipes* (Mart.) Solms-Laubach (Sacco et al. 2013).

The symptoms caused by *P. rubra* feeding injury to cranberry vines reported in our study are similar to those reported by Sirrine and Fulton (1914). This previous study described the injury by *P. rubra* feeding to cranberries based on field observations and not on controlled experiments; thus, the study by Sirrine and Fulton (1914) could not with certainty associate the observed injury to *P. rubra*. In our density-manipulative studies, *P. rubra* feeding caused leaves of cranberry vines to turn brown, which resembles the appearance of vines when they die after being broken. As expected, our results showed significant effects of *P. rubra* density on vine health. In general, higher insect densities resulted in a reduced proportion of healthy vines and an increased proportion of stressed (injured) vines. These results were consistent between our cage experiments in the screen-house and field (2015‒2016) and the open field experiment (2017). Our open field experiment correlated *P. rubra* densities in late July to early August with injury to vines, which coincides with the presence of later nymphal stages and early adults in the field (Sirrine and Fulton 1914, C.R.-S. unpublished data).

The injury by *P. rubra* to cranberry vines is similar to the injury caused by hemipteran pests to other crops. For example, feeding injury by *Anasa tristis* (DeGeer) (Hemiptera: Coreidae) to cucurbits causes the foliage to wilt, blacken, and die (Capinera 1999), which is directly proportional to the density of *A. tristis* (Woodson and Fargo 1992). Unlike *A. tristis* that can transmit a bacterium that causes cucurbit yellow vine disease (Bruton et al. 2003, Pair et al. 2004), *P. rubra* is not known to transmit a disease in cranberries, indicating that the observed injury is likely caused by direct feeding of *P. rubra*. The injury caused by *P. rubra* to cranberries is different from that of other aboveground pests, which makes it easy to diagnose. Although the exact site (plant tissue) of feeding that causes this injury remains unknown because of the systemic nature of the symptoms, we assume that the reported *P. rubra* injury to vines is caused directly by the insects sucking fluids from the phloem and xylem of, and/or indirectly by injection of toxic substances to, the cranberry stems. Additional research is needed to confirm this assumption. Feeding by piercing-sucking herbivores is known to alter plant nutritional composition (e.g., Tobih et al. 2002, Xiao and Fadamiro 2010, Cherry et al. 2013), which in turn could affect the preference of these herbivores for host plants (e.g., Mace and Mills 2015, Sétamou et al. 2016). In the case of *P. rubra*, however, we found no evidence that feeding injury changes the composition of macro- (P, K, Ca, Mg, and S) or microelements (Mn, Zn, Fe, Cu, B, Na, and Al) in cranberry vines.

Injury to vines caused by *P. rubra* feeding can result in a reduction of fruit numbers and mass due to dwarfed or shriveled (rotten) berries. Although the effect on fruit was not always significant, increased *P. rubra* densities generally resulted in a reduced number of healthy fruit and lowered fruit mass. In our open field experiment, higher *P. rubra* densities in the month of July were correlated with less fruit. As indicated before, this time of the year was also critical for *P. rubra* injury to cranberry vines. It is thus likely that injury to vines due to feeding by nymphs caused the observed reduction in fruit production. Unlike other hemipteran pests that feed upon and prefer reproductive structures such as the brown marmorated stink bug [*Halyomorpha halys* Stål (Hemiptera: Pentatomidae)] (Rice et al. 2014), we found little evidence that *P. rubra* causes direct injury to cranberry fruits because injured fruit showed little to no signs of feeding by insects. A few factors could have contributed to some inconsistencies obtained in our caged studies for the effects of *P. rubra* on fruit quality and quantity. For example, a significant decline in the proportion of healthy fruit was found with increasing insect densities in screen-house cages, whereas increasing *P. rubra* densities only lowered fruit mass significantly in field cages. The length of the former study was longer, which might have allowed for fruit to rot, whereas in the latter study, the vine to insect ratio was lower. Thus, the length of injury and number of *P. rubra* in relation to vines will likely determine the extent of the negative effect on fruit yield.

Not surprisingly, we found a positive correlation between the proportion of injured vines and *P. rubra* density in our open field experiment. In contrast, *P. rubra* density was negatively correlated with fruit yield. Although these correlations were significant, it is worth noting that the variation explained by both regression models was low (<25%), which indicates that abiotic (i.e., weather conditions, water availability, etc.) and/or biotic (i.e., other insect pests, diseases, etc.) factors other than *P. rubra* feeding injury affected vine health and fruit production in cranberry beds. Hall and Teetes (1982) indicated that sorghum grain was most susceptible to attack by *Leptoglossus phyllopus* (L.) (Hemiptera: Coreidae) adults in the early stage of development. Similarly, we found that cranberries were more susceptible to *P. rubra* injury in the early stages of fruit development since insect densities in July, and not in August and September, were correlated with crop injury.

An objective of this study was to extrapolate the data on injury to vines and fruit to recommend an economic or action threshold for *P. rubra* on cranberries. *Phylloscelis rubra* feeding affected vine health and fruit quality (mass) and quantity; however, our data show that not all insect densities resulted in significant injury to cranberries. In cage experiments in the field, we found that densities as low as one to two *P. rubra* per vine can result in vine injury and a reduction in fruit quality. Sirrine and Fulton (1914) also noted that feeding by one *P. rubra* on new growth could cause cranberry vines to wilt and discolor. Xiao and Fadamiro (2010) reported that more than two *Leptoglossus zonatus* (Dallas) (Hemiptera: Coreidae) individuals per fruit is needed to reduce satsuma mandarin (*Citrus unshiu* Marcovitch) fruit mass. Similarly, Hall and Teetes (1982) set the damage threshold at six *L. phyllopus* adults per panicle to cause significant reduction in the yield of green sorghum. However, data collected from cage studies in which insect movement is restricted may not necessarily provide an accurate estimation of an economic or action threshold. To address this concern, we conducted an open field experiment. Although several factors could have influenced our field data, in general, our open field experiment corroborated results from our cage experiments. In an open field experiment, we showed that even low densities of *P. rubra* (i.e., <10 individuals per sweep set) can significantly increase vine injury and reduce cranberry yield. Thus far, it is difficult to propose with accuracy an economic or action threshold for *P. rubra* in cranberries due to several factors, including lack of effective monitoring methods (farmers do not like to use sweep nets during fruit development due to potential injury to the crop) and the inability to predict its movement within and among bogs. To establish an economic injury level (lowest insect density that will result in economic loss) for *P. rubra*, future field studies need to test the effects of an insecticide treatment at various densities per bed (e.g., at low [<10], intermediate [10–20 and 20–30], and high [30–40] numbers per sweep net) on cranberry vine injury and yield.

In conclusion, farmers are constantly dealing with new and emerging pests that can disrupt existing integrated pest management practices. In New Jersey, *P. rubra* is an emerging insect pest of cranberries. This study documents for the first time the effects of *P. rubra* density on crop injury in cage studies and an open field experiment. In these cage and open field studies, we showed that even low densities of *P. rubra* could result in the discoloration and death of vines, which may cause reduced fruit quality and yield. Although we established that infestation levels of <10 *P. rubra* per sweep net could result in crop injury and potentially economic losses, action thresholds for this pest still need to be validated at large farm scales. Because the type of injury caused by *P. rubra* to vines can be observed aerially and the number of bugs are positively correlated with vine injury, unmanned aerial vehicles or drones (Iost Filho et al. 2020) could be used as an alternative method to sweep nets to monitor the extent of cranberry injury while minimizing disturbance to the crop.
